# Wnt/β-catenin signaling pathway may regulate the expression of angiogenic growth factors in hepatocellular carcinoma

**DOI:** 10.3892/ol.2014.1828

**Published:** 2014-01-27

**Authors:** BO QU, BING-RONG LIU, YA-JU DU, JING CHEN, YAN-QIU CHENG, WEI XU, XIN-HONG WANG

**Affiliations:** Department of Gastroenterology, The Second Affiliated Hospital of Harbin Medical University, Harbin, Heilongjiang 150086, P.R. China

**Keywords:** hepatocellular carcinoma, RNA interference, β-catenin, signaling pathway, angiogenesis factor

## Abstract

The Wnt/β-catenin signaling pathway plays a key role during hepatocellular carcinoma (HCC) genesis and development. The present study aimed to investigate the effects of the Wnt/β-catenin signaling pathway on the expression of angiogenic growth factors involved in HCC. The HCC HepG2 cell line was transfected with small interfering RNA (siRNA) against β-catenin. After 72 and 96 h, protein was extracted and the expression levels of β-catenin, matrix metalloproteinase (MMP)-2, MMP-9, vascular endothelial growth factor (VEGF)-A, VEGF-C and basic fibroblast growth factor (bFGF) were detected by western blot analysis. β-catenin protein expression was inhibited at both time points. Notably, MMP-2, MMP-9, VEGF-A, VEGF-C and bFGF protein expression levels decreased at 72 h and then increased at 96 h after transfection. Our results demonstrated that in HCC cells, the Wnt/β-catenin signaling pathway may regulate the protein expression of the angiogenic factors, MMP-2, MMP-9, VEGF-A, VEGF-C and bFGF. These proteins were downstream of β-catenin signaling and were also regulated by other factors. In conclusion, the Wnt/β-catenin signaling pathway may contribute to the regulation of HCC angiogenesis, infiltration and metastasis through regulating the expression of these angiogenic factors.

## Introduction

The cytoplasmic protein, β-catenin, is a central molecule in the Wnt signaling pathway and plays a key role in the genesis and development of tumors. The Wnt/β-catenin signaling pathway has been shown to be deregulated in hepatocellular carcinoma (HCC) (1–3), gastric cancer (3), breast cancer (4), and colon cancer (3,5,6). When β-catenin phosphorylation and ubiquitin-dependent degradation are inhibited, β-catenin concentrates in the cytoplasm and forms a complex with the transcription factor, lymphoid enhancing factor-1 (LEF_1_)/T-cell factor (TCF), which is subsequently transported into cell nuclei. This transcription complex activates the expression of downstream target genes, resulting in abnormal cell proliferation and cell carcinogenesis (6). To date, several target genes of this signaling pathway have been identified, including c-jun, c-fos, c-myc (7,8), cyclins (7,9), survivin (7) and peroxisome proliferator-activated receptor-γ. Further research on the target genes of the Wnt/β-catenin signaling pathway is required to increase our understanding of the role of this pathway in the genesis and development of tumors.

Research on tumor angiogenic factors was initiated in 1968 (10). To date, a number of crucial angiogenic factors have been isolated, including vascular endothelial growth factor (VEGF), tissue factor, matrix metalloproteinase (MMP), fibroblast growth factor (FGF) and tumor necrosis factor. These factors play crucial roles in tumor angiogenesis, infiltration and metastasis (11–16). Understanding the roles of these angiogenic factors is an active area of research.

Previous studies have identified that the Wnt/β-catenin signaling pathway is involved in the regulation of angiogenic factors. It was reported that the β-catenin pathway regulated the expression of VEGF, MMP-7 and other factors in human colorectal cancer (17). Additionally, in damaged lungs, a correlation between β-catenin overexpression and increased VEGF expression levels has been demonstrated (18). Moreover, it has been reported that MMP-3 is a direct transcriptional target and a necessary contributor of the Wnt/β-catenin signaling pathway (19). Furthermore, Wnt/β-catenin signaling influenced the expression of MMP-2 and MMP-9 in T cells (20). However, confirmation of the possible association between the Wnt/β-catenin signaling pathway and the expression of angiogenic factors has not yet been fully elucidated.

HCC has a strong tendency to infiltrate and metastasize, and angiogenic factors have key roles in tumor angiogenesis, infiltration and metastasis. Therefore, in the present study, β-catenin expression was silenced using RNA interference (RNAi) technology in order to examine the association between the Wnt/β-catenin signaling pathway and angiogenic factors in HCC. We aimed to further elucidate the role of the Wnt/β-catenin signaling pathway in the pathogenesis of HCC.

## Materials and methods

### Materials

The HCC HepG2 cell line was purchased from the cell bank of the Chinese Academy of Sciences (Guangzhou, China). Lipofectamine™ 2000 was purchased from Invitrogen Life Technologies (Carlsbad, CA, USA). Small interfering RNA (siRNA) directed against β-catenin was designed and synthesized by the Shanghai GenePharma Co., Ltd. (Shanghai, China). The siRNA sequences were as follows: Sense, 5′-GGGUUCAGAUGAUAUAAAUTT-3′; and antisense, 5′-AUUUAUAUCAUCUGAACCCAG-3′. Dulbecco’s modified Eagle’s medium (DMEM) with high glucose was purchased from the Shanghai Hecoly Automatic Control Equipment Co., Ltd. (Shanghai, China) and fresh fetal calf serum (FCS) was purchased from TBD Biotechnology Corp. (Tianjin, China). The primary antibodies used were mouse anti-β-catenin, mouse anti-β-actin, rabbit anti-MMP-2, rabbit anti-MMP-9, mouse anti-VEGF-A, goat anti-VEGF-C and mouse anti-bFGF. The secondary antibodies of horseradish peroxidase (HRP)-conjugated goat anti-rat, goat anti-rabbit and rabbit anti-goat were used. All antibodies were purchased from Santa Cruz Biotechnology, Inc. (Santa Cruz, CA, USA).

### Cell culture

HepG2 cells were plated in culture flasks and were cultured in DMEM supplemented with 10% (vol/vol) fresh FCS at 37°C in a humidified atmosphere with 5% CO_2_. Cells were replated when they reached confluency and digested with 0.25% trypsin for 1 min in order to maintain the cell line.

### Transient transfection

A total of 3×10^5^ cells were plated in six-well plates in triplicate and grown to ~30–50% confluency. For transfection, 500 μl of siRNA in Lipofectamine 2000 was added to each well containing cells and 2 ml of DMEM without FCS, and plates were gently rocked back and forth. After 6 h of transfection, the media were replaced with DMEM containing 10% (vol/vol) FCS. The cells were harvested after 72 and 96 h, and mRNA and proteins were isolated for further analyses. Control groups without the addition of transfection reagents were also included in this study. All experiments were performed in triplicate and representative results were reported.

### Western blot analysis

Cells were lysed in radioimmunoprecipitation assay buffer 72 and 96 h after transfection for protein isolation (Beyotime, Shanghai, China). Protein concentrations were determined using the bicinchoninic acid assay (Beyotime). Protein samples were electrophoresed on 10% SDS-PAGE gels (β-catenin, MMP-2, MMP-9 and VEGF-C) or 15% SDS-PAGE gels (VEGF-A and bFGF) and transferred onto polyvinylidene difluoride membranes. The membranes were blocked by incubation in phosphate-buffered saline (PBS) containing 5% skimmed milk at 37°C for 1 h and then incubated with specific primary antibodies (1:200 dilution) at 4°C overnight. The membranes were rinsed three times with PBS, followed by incubation with HRP-conjugated secondary antibodies (1:5,000 dilution) at 37°C for 1 h. Membranes were then rinsed three times with PBS and developed in 3,3′diaminobenzidine. When protein bands were visible on the membranes, color development was discontinued and membranes were rinsed in distilled water. Images were captured by the Gel Imaging system (Tanon Science and Technology Co., Shanghai, China) and images were analyzed by Quantity One software (Bio-Rad Laboratories, Berkeley, CA, USA). The quantitative results of gray-scale analysis were used for the statistical analysis.

### Statistical analysis

Data were compared by Student’s t-test. P<0.05 was considered to indicate a statistically significant difference.

## Results

### Western blot analysis

**Figure 1:**
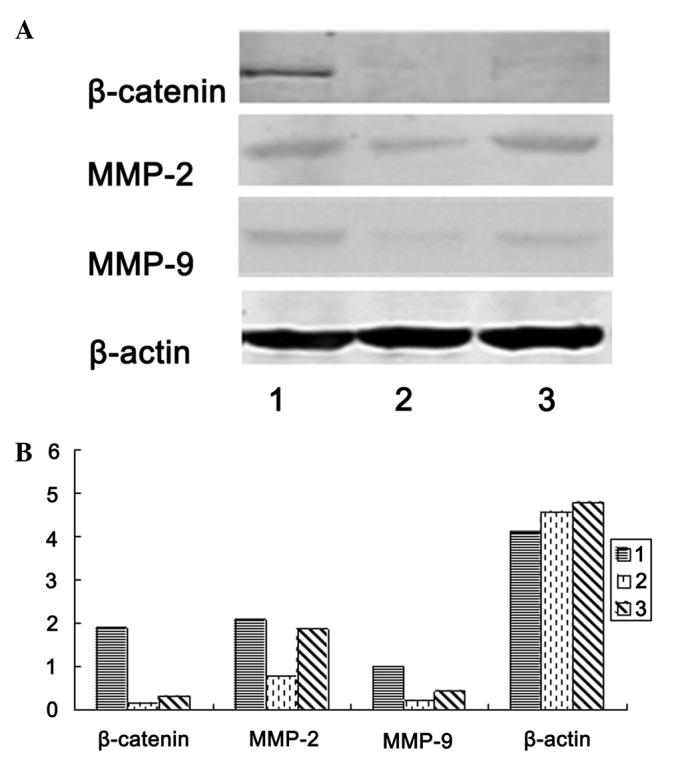
Expression of β-catenin, MMP-2, MMP-9 and β-actin after knockdown of β-catenin by small interfering RNA transfection in hepatocellular carcinoma HepG2 cells. (A) Western blotting of the protein expression in the control group (1), 72 h after transfection (2) and 96 h after transfection (3). (B) Protein expression in the control group (1), 72 h after transfection (2) and 96 h after transfection (3). Y-axis is the quantitative results of gray-scale analysis. MMP, matrix metalloproteinase.

**Figure 2:**
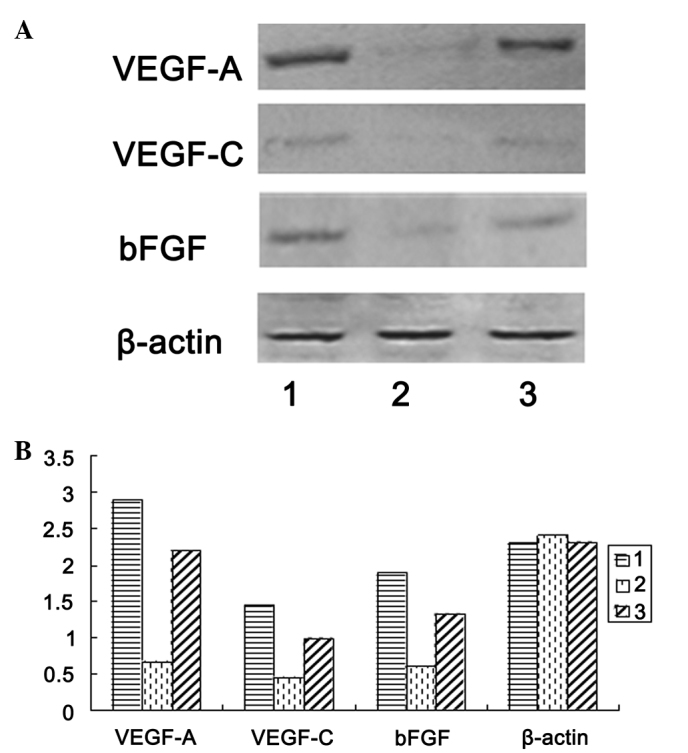
Expression of VEGF-A, VEGF-C, bFGF and β-actin after knockdown of β-catenin by small interfering RNA transfection in hepatocellular carcinoma HepG2 cells. (A) Western blotting of the protein expression in the control group (1), 72 h after transfection (2) and 96 h after transfection (3). (B) Protein expression levels in the control group (1), 72 h after transfection (2) and 96 h after transfection (3). Y-axis is the quantitative results of gray-scale analysis. VEGF, vascular endothelial growth factor; bFGF, basic fibroblast growth factor.

Protein expression was assessed by western blotting at 72 and 96 h after the transfection of siRNA against β-catenin into HCC HepG2 cells. Our results demonstrated that the expression levels of β-catenin were decreased after 72 and 96 h, although the levels were slightly higher after 96 h than those at 72 h (t=4.43; P<0.05). MMP-2 and -9 protein expression was inhibited at 72 h, but that of MMP-2 had returned to normal levels after 96 h (t=0.68; P>0.05). MMP-9 expression levels were also increased at 96 h, but were less than the levels observed in the control group (t=19.74; P<0.01) (Fig. 1). The expression of VEGF-A and -C was inhibited at 72 h and, although expression increased at 96 h, the levels remained lower than those of the control group (t=10.36 and t=15.31, respectively; P<0.01). bFGF expression showed a pattern that was similar to that observed for VEGF and MMP, with inhibition at 72 h and increased expression at 96 h (t=44.11; P<0.01) (Fig. 2).

In conclusion, following the transfection of siRNA against β-catenin into HCC HepG2 cells, β-catenin protein expression was inhibited after 72 and 96 h. MMP-2, -9, VEGF-A, -C and bFGF expression levels were also decreased at 72 h, but began to recover to normal levels at 96 h. Based on these observations, we hypothesized that the expression of these important factors was regulated through the Wnt/β-catenin signaling pathway.

## Discussion

RNAi, first reported by Fire *et al* in 1998 (21), is a gene silencing technique at the post-transcriptional level caused by the introduction of a double-stranded RNA, which induces the degradation of mRNA containing specific homologous sequences (20). To date, RNAi has been successfully applied to the study of gene functions and the associations between the upstream and downstream factors in signaling pathways. RNAi may also potentially be applied in future tumor therapies. The present study identified that the protein expression of β-catenin was inhibited at 72 and 96 h after the transfection of siRNA against β-catenin into HCC HepG2 cells.

Following knockdown of β-catenin in HCC HepG2 cells for 72 h, the protein expression levels of MMP-2, -9, VEGF-A, -C and bFGF also decreased. These findings indicated that the Wnt/β-catenin signaling pathway can regulate the expression of these proteins and that they are downstream target proteins of β-catenin signaling. However, the underlying mechanisms involved in this regulation have not yet been fully determined. Previous studies have reported that the Wnt/β-catenin signaling pathway acted directly on the MMP promoter through LEF_1_/TCF binding in T cells (20), but this regulatory mechanism has not been reported in HCC and is thus the subject of future investigations in our laboratory.

MMPs promote angiogenesis and contribute to tumor infiltration and metastasis not only through degradation of the extracellular matrix and vascular basilemma, but also through the active regulation of transforming growth factor-β, bFGF, VEGF and other important signaling molecules. The VEGF family regulated the formation of blood vessels and lymphatic vessels, vascular permeability and endothelial cell survival (23). Moreover, angiogenesis and lymphangiogenesis may promote tumor metastasis. bFGF expression has been correlated with the promotion of cancer cell proliferation and tumor angiogenesis. Additionally, bFGF expression can regulate the activities of collagenase, protease, urokinase-type plasminogen activator and integrins. bFGF also stimulated the secretion of VEGF, another key regulatory factor with synergistic activities. Thus, the Wnt/β-catenin signaling pathway contributed to HCC angiogenesis, infiltration and metastasis through regulating the expression of MMP-2, -9, VEGF-A, -C and bFGF.

In addition, MMP-2, -9, VEGF-A, -C and bFGF protein expression levels increased after blocking β-catenin expression for 96 h in HepG2 cells. These results demonstrated that the Wnt/β-catenin signaling pathway is not the only factor regulating the expression of these proteins and that a number of other factors are also involved in their regulation. These findings were consistent with other previous studies. Several studies have reported that the STAT3 signaling pathway influenced tumor angiogenesis, infiltration and metastasis by regulating the expression of VEGF, MMPs or bFGF in pancreatic cancer (24), colorectal cancer (25), gastric cancer (26), HCC (27) and several other types of tumors. In fibrosarcoma (28) and colorectal cancer (29) cells, the mitogen-activated protein kinase (MAPK) signaling pathway inhibited the expression of VEGF, bFGF and STAT3, and the p38 MAPK signaling pathway mediated VEGF expression in bone marrow mesenchymal stem cells (29). In addition, β2-glycoprotein I inhibited the angiogenesis induced by VEGF and bFGF through the activity of its amino terminal domain (31). In prostate cancer, MMP-2 and -9 expression was regulated by the androgen receptor signaling pathway and was associated with tumor invasion (32). In liver cancer, MMP-2 and -9 expression and activities were upregulated and downregulated by recombinant N-terminal of Sonic Hedgehog (SHH) and the SHH signaling inhibitor (33). Semaphorin 6A regulated angiogenesis by modulating VEGF signaling (34). Collectively, the multiple pathways that have been reported to participate in the regulation of these angiogenic factors emphasized the complicated regulation of tumor angiogenesis, invasion and metastasis.

In conclusion, this study demonstrated that the Wnt/β-catenin signaling pathway contributed to HCC angiogenesis, infiltration and metastasis through regulating the expression of angiogenic factors. The results of the present study require further validation in other cell lines and in animal studies. In addition, the target genes of the Wnt/β-catenin signaling pathway and the regulation of angiogenic factors have not yet been fully elucidated. Our results aid us to better understand the complex network underlying HCC pathogenesis and provide the basis and methods for multiple aspects of HCC research, including prevention, diagnosis and treatment. With the continual progression of HCC research, a number of concepts will be updated and bring us closer to curing HCC.
